# Characterization of antipsychotic utilization before clozapine initiation for individuals with schizophrenia: an innovative visualization of trajectories using French National Health Insurance data

**DOI:** 10.1017/S2045796023000732

**Published:** 2023-09-19

**Authors:** Edouard-Jules Laforgue, Marion Istvan, Anicet Chaslerie, Pascal Artarit, Geneviève Vallot, Pascale Jolliet, Marie Grall-Bronnec, Caroline Victorri-Vigneau

**Affiliations:** 1 CHU Nantes, Service de Pharmacologie Clinique, Nantes Université, Nantes, France; 2 CHU Nantes, INSERM, MethodS in Patient-Centered Outcomes and HEalth ResEarch, SPHERE, INSERM, Methods in Patient-Centered Outcomes and Health Research, SPHERE, Nantes Université, Univ Tours, Nantes, France; 3 Medical Department, Regional Health Insurance Pays de la Loire, Nantes, France; 4 CHU Nantes, UIC Psychiatrie et Santé Mentale, Nantes Université, Nantes, France

**Keywords:** antipsychotics, clozapine, pharmacoepidemiology, state sequence analysis

## Abstract

**Aims:**

Despite recommendations to initiate clozapine after two unsuccessful trials of antipsychotics, clozapine is underprescribed and initiated too late. The aim of this study was to describe different antipsychotic treatment sequences in the 36 months before the initiation of clozapine and to characterize clusters of treatment trajectories.

**Methods:**

Using the French National Health Insurance database, a historical cohort study of the population in an area in western France was performed. The data from all new users of clozapine with a diagnosis of schizophrenia or schizoaffective disorder in the period of 2017–2018 were evaluated. All outpatient reimbursements for antipsychotics during the 36 months before clozapine initiation were analysed. Successive reimbursements for identical treatments were grouped into treatment trials (TTs), and different trajectories were clustered using a state sequence analysis.

**Results:**

The results showed 1191 TTs for 287 individuals. The mean number of TTs per individual was 3.2. Risperidone, aripiprazole and haloperidol were the main treatments delivered. The frequencies of antipsychotics used differed between monotherapies and combination therapies. A three-cluster typology was identified: one cluster (*n* = 133) of ‘less treated’ younger individuals with fewer TTs and shorter TT durations; a second cluster (*n* = 53) of ‘more treated’ individuals with higher numbers of TTs and combinations of antipsychotics; and a third cluster (*n* = 103) of ‘treatment-stable’ older individuals with longer TT durations.

**Conclusions:**

The results indicate that the median number of TTs during the 36 months before clozapine prescription was higher than the two recommended. The different trajectories were associated with individual characteristics and treatment differences, suggesting that additional studies of clinical parameters are needed to understand barriers to clozapine prescription.

## Introduction

### Clozapine: an underused drug

Compared to other antipsychotics, clozapine is the only drug with better efficacy in the treatment of schizophrenia and reduction of mortality in these individuals (Huhn *et al.*, 2019; Tiihonen *et al.*, 2009). Due to the risk of agranulocytosis, clozapine prescription is restricted to individuals with treatment-resistant schizophrenia (TRS), and its prescription is carried out with mandatory (with slight variations across different countries) haematological monitoring.

Among patients with schizophrenia, 20–30% are considered to have TRS (Moore *et al.*, 2007), generally defined by the absence of clinical improvement following two adequate trials of different antipsychotics (NICE, 2014). In this condition and with adequate plasma levels (>350 ng/ml), clozapine is effective in 60% of individuals (Iqbal *et al.*, 2003; Kronig *et al.*, 1995; Schulte, 2003). According to these data, Bogers et al. estimated that 12–18% of schizophrenic individuals would be expected to use clozapine (Bogers *et al.*, 2016); however, the actual percentage is far lower for several reasons related to characteristics of clinicians, patients and institutions as well as healthcare systems (Farooq *et al.*, 2019; Verdoux *et al.*, 2018). The rate of clozapine prescription is estimated to be 2% in the USA (Bogers *et al.*, 2016), a rate steadily declining since 2014 (Warnez and Alessi-Severini, 2014), and to be 3% in France (Mouaffak *et al.*, 2016). Additionally, the rate of antipsychotic polypharmacy (APP) has reached up to 40% (Cotes *et al.*, 2018) despite clinical practice recommendations emphasizing monotherapy (Galletly *et al.*, 2016; Hasan *et al.*, 2012; Lehman *et al.*, 2004; NICE, 2014).

### Clozapine: a latecomer for TRS treatment

Evidence of the efficacy of clozapine from the CATIE and CUtLASS studies suggests that clozapine should be prescribed after two, not three, ineffective trials of antipsychotics, regardless of whether these antipsychotics are first- or second-generation antipsychotics (Carpenter and Buchanan, 2008). In 2004, a retrospective study of almost 2800 individuals from the Auckland and Northland regions of New Zealand found an average delay of 9.7 years and an average number of 3.5 trials of antipsychotics between the onset of schizophrenia and the initiation of clozapine (Wheeler, 2008). Health policies related to clozapine reimbursement in New Zealand were associated with a reduction in the delay of the first clozapine prescription to under a year (Wheeler *et al.*, 2014), thus approaching the recommendations to prescribe as soon as possible (Galletly *et al.*, 2016). Despite these encouraging results, other data from the United Kingdom (UK) and Denmark reflect also excessive delay before initiation. In a study by Howes et al. in 2012, for 149 UK individuals, the mean duration before clozapine prescription was 4 years, and over 5 trials of antipsychotics were attempted beforehand (Howes *et al.*, 2012). According to Danish national registry data, Nielsen et al. found a longer duration of illness before clozapine prescription in 2003 than in 1996 (Nielsen *et al.*, 2012). Thus, when prescribed, clozapine is initiated too late in the TRS trajectory.

### The need to characterize treatment trajectories before clozapine initiation

Delayed and consecutive unsuccessful antipsychotic trials before clozapine prescription correspond to a real loss for individuals given that an efficient treatment response is a major prognostic factor (Agid *et al.*, 2007; Alphs *et al.*, 2016; Shah *et al.*, 2018; Taylor *et al.*, 2003). While the onset of schizophrenia is generally in adolescence and early adulthood, international trends in clozapine prescription reveal peaks of prescription at ages of 40–59 years, with a heterogeneity of profiles concerning age and sex across different countries (Bachmann *et al.*, 2017). There is no algorithm to guide successive antipsychotic trials before clozapine (Kahn *et al.*, 2018), and information on treatment trajectories (*i.e.*, what has been prescribed) and individual characteristics associated with a late initiation of clozapine is scarce. In clinical practice, data about individuals’ treatment trajectories can also be missing, notably due to memory bias and/or lack of sharing of medical files. In France, data from the *Système National d’Informations Inter-Régimes de l’Assurance Maladie* (SNIIRAM), which provides outpatient healthcare reimbursements (including drug deliveries), and the *Programme de Médicalisation des Systèmes d’Information* (PMSI), which contains data about hospitalizations (hospitalization dates and diagnostic codes according to the International Classification of Diseases, 10th edition [ICD-10]), are compiled in a national healthcare database, the *Système National des Données de Santé* (SNDS). The SNDS covers more than 98% of French residents from birth (or immigration) to death (or emigration) and contains individualized and anonymized data (Bezin *et al.*, 2017; Scailteux *et al.*, 2019). Because of its comprehensiveness, this database can be used to identify drug trajectories. Thus, the aim of the present study was (i) to describe different antipsychotic treatment trajectories in the 36 months before the initiation of clozapine in individuals with schizophrenia or schizoaffective disorder (SSAD) and (ii) to identify and characterize different clusters of individuals according to their treatment trajectories.

## Materials and methods

### Study overview

We performed a historical cohort study of individuals diagnosed with SSAD who were prescribed clozapine to identify antipsychotic trials in the 36 months beforehand using a subset of SNDS data (data from Pays de la Loire, an area in western France). The population in this area covered by the SNDS was approximately 3.7 million. We selected all individuals with an SSAD diagnosis newly prescribed clozapine in the period of 2017–2018 for analysis. The 2-year inclusion period combined with the 5-year depth of the SNDS allowed us to analyse data over a period of at least 36 months for each subject. Using this data, we analysed all outpatient reimbursements for psychotropic drugs, including antipsychotic drugs.

The data extracted from the claims database were as follows:

- *Individual characteristics*: age at inclusion (index date of clozapine initiation); sex (assigned at birth); the presence of Universal Complementary Health Care Coverage (*Couverture maladie universelle complementaire*, CMU-c), an indicator of low income; registratered diagnosis and its date in the list of Chronic Disease Scheme (CDS) beneficiaries (*Affection Longue Durée*); and hospitalization (in a psychiatric or somatic ward) during the follow-up period
- *Treatment characteristics*: for each psychotropic treatment reimbursement, the drug, date of delivery and quantity delivered were extracted (due to SNDS-specific settings, data on treatments delivered during hospitalizations are not available)
- *Prescriber characteristics*: number of distinct prescribers for psychotropic medications and type of service (public health system or private practitioner)

Data were extracted and analysed according to data protection laws (*Commission nationale de l’informatique et des libertés*, CNIL).

### Study population

- *Individuals with SSAD diagnosis* were identified using diagnostic codes reported in the CDS registry or for hospitalizations according to the ICD-10 codes of F20 (schizophrenia) and F25 (schizoaffective disorder).
- *Among these individuals, an individual with clozapine initiation* was defined as an individual without reimbursement for clozapine in the 12 months before the first reimbursement of clozapine during the study period (1 January 2017–31 December 2018).
- We analysed the consecutive different effective antipsychotic treatments sequences in the 36 months before the initiation of clozapine.

Psychotropic medications were considered ‘*effective antipsychotic treatments*’ when

- the indication of ‘schizophrenia’ and/or ‘long-term treatment of chronic psychosis’ was mentioned in the French marketing authorization (amisulpride, aripiprazole, chlorpromazine, clozapine, cyamemazine, flupentixol, fluphenazine, haloperidol, levomepromazine, loxapine, olanzapine, paliperidone, pimozide, pipotiazine, quetiapine, risperidone, sulpiride, zuclopenthixol) and
- when a delivery with an average daily dose ≥80% of the defined daily dose (DDD) was mentioned in the summary of product characteristics. The database does not provide information on the individual prescribed dosage and duration delivered. In France, unless otherwise stated on the prescription, medications are delivered for a period of 28 days. The assumption was made that a delivery corresponded to 28 days of treatment. The average daily dose per delivery was thus calculated by dividing the total antipsychotic dose by 28. The cut-off of 80% of the DDD was an adaptation of the accepted cut-off to define nonadherence (Velligan *et al.*, 2009) and can, with a reasonable margin of error, distinguish an adequate antipsychotic trial from an insufficient dosage of antipsychotic (Howes *et al.*, 2017).

Other psychotropic drugs, including antipsychotics with an average daily dose <80% of the DDD (i.e., noneffective antipsychotic treatments), were not considered in the primary analysis but are described as ‘*other psychotropic drugs*’ in supplementary materials.

### Data management

**Figure 1.**
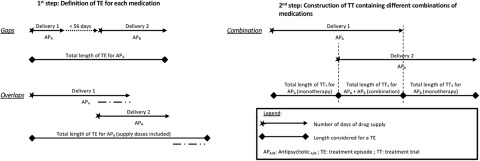
Illustration of the method for the definition of effective treatment episodes (TEs) and treatment trials (TTs).

The data of consecutive deliveries of effective antipsychotic treatments in the 36 months before the initiation of clozapine were managed in two steps. First, treatment episodes (TEs) were constructed for each antipsychotic drug that was reimbursed for at least two successive deliveries with a maximum gap of 56 days between these two deliveries (after the removal of the number of days of hospitalization (if present) and taking into account overlapping periods for the same antipsychotic). We considered the absence of a new delivery of the antipsychotic over a period of 2 × 28 days from the previous delivery to constitute an interruption of treatment. Second, TEs were aggregated to define final distinct treatment trials (TTs), taking into account the combination of antipsychotic treatments received simultaneously (Fig. 1).

### Outcomes

The primary outcome was the description of TEs and TTs during the 36 months preceding the initiation of clozapine. Secondary outcomes were (i) the identification of different clusters of TTs trajectories (‘therapeutic pathways’) before the initiation of clozapine and (ii) the characterization of these different clusters.

### Statistical analysis

Categorical data are expressed as counts and percentages, and continuous data are expressed using the mean and standard deviation, 95% confidence interval, or median and interquartile range (IQR).

State sequence analysis was performed using the R package TraMineR v2.0-12 (Gabadinho *et al.*, 2011). This method was used to assess consecutive TTs during the 36 months preceding the initiation of clozapine. Each TT was categorized as different compared with the preceding TT. Sequences were composed of 36 states corresponding to the 36 months. Each state was defined according to the situation that was most representative of the month. We computed pairwise distances using the optimal matching method, in which the dissimilarity between the sequences was defined as the minimal cost to change from one sequence to another.

We performed agglomerative hierarchical clustering with the Ward method to identify clusters of similar sequences. The number of clusters was chosen using the fall in inertia and two quality criteria: the weighted average silhouette width (ASWw) and Hubert’s C index (HC). The patterns of the clusters identified were represented using transversal state distribution plots. Comparisons among the clusters were performed using ANOVA or the Kruskal‒Wallis test for continuous variables and the chi-squared test for categorical variables. Sensitivity analyses were performed to take into account the duration of SSAD using the time since its CDS registration as a proxy: 3–10 or >10 years.

Analyses were performed using SAS-Enterprise Guide version 7.1 and R software version 3.6.1 (R Core Team, 2020).

## Results

Of the 348 individuals in the *Pays de la Loire* area with clozapine initiation and an SSAD diagnosis entered in the SNDS database, 23 were excluded because they were considered misdiagnosed, as they had at least one delivery of antiparkinsonian dopaminergic medication during the 36 months but no antipsychotic reimbursement. In this population (*n* = 325), data from 287 individuals with at least one effective TT were analysed.

The mean age was 42.9 (16.4) years, and the majority of individuals were male (60.6%). A total of 43.9% were covered by the CMU-c (i.e., national health coverage for low-income people). The mean elapsed time between CDS registration of SSAD and the initiation of clozapine was 13.0 years (minimum: −2 years; maximum: 49 years). For 68% of the individuals (195/287), at least one hospitalization occurred during the follow-up period (143 in a psychiatric ward and 114 in a medicine ward).

### Primary outcome

The 287 individuals analysed received 8891 reimbursements for effective antipsychotic treatments in 1166 TTs. The median number of different successive TTs per individual (i.e., a TT different from the preceding TT) was 3 [IQR: 1–5; maximum = 14]. Detailed results concerning the frequencies of antipsychotics as TTs in the sample are presented in Table 1.

**Table 1.** Distributions of the main antipsychotics in treatment trials

	Total individuals (*n* = 287)			
Antipsychotic	Proportion of individuals with at least 1 TT with the AP (% (*n/287*)	Detailed proportions of monotherapy *only* for individuals who received this antipsychotic (% (*n*))	Detailed proportions of combinations *only* for individuals who received this antipsychotic (% (*n*))	Detailed proportions of monotherapy and combinations for individuals who received this antipsychotic (% (*n*))
Risperidone	44.6 (128)	37.5 (48/128)	31.3 (40/128)	31.3 (40/128)
Aripiprazole	34.1 (98)	26.5 (26/98)	37.8 (37/98)	35.7 (35/98)
Haloperidol	29.6 (85)	28.2 (24/85)	31.8 (27/85)	40.0 (34/85)
Olanzapine	26.5 (76)	25.0 (19/76)	39.5 (30/76)	35.5 (27/76)
Quetiapine	24.7 (71)	26.8 (19/71)	38.0 (27/71)	35.2 (25/71)
Paliperidone	19.2 (55)	29.1 (16/55)	36.4 (20/55)	34.5 (19/55)
Amisulpride	16.7 (48)	12.5 (6/48)	50.0 (24/48)	37.5 (18/48)
Cyamemazine	17.8 (51)	3.9 (2/51)	80.4 (41/51)	15.7 (8/51)
Loxapine	16.7 (48)	10.4 (5/48)	77.1 (37/48)	12.5 (6/48)
Others	15 (43)	32.6 (14/43)	48.8 (21/43)	18.6 (8/43)

TT = treatment trial.

The most frequent antipsychotics prescribed for the whole sample were risperidone (45%), followed by aripiprazole (34%) and haloperidol (30%). Risperidone was the main antipsychotic prescribed as monotherapy (38%) but least frequently included in APP. Considering the patients with APP, 182 (63%) individuals in the whole cohort had a combination of two antipsychotics, 52 (18%) had a combination of three antipsychotics and 8 (3%) had a combination of four antipsychotics. Cyamemazine and loxapine were most frequently included in APP.

**Figure 2.**
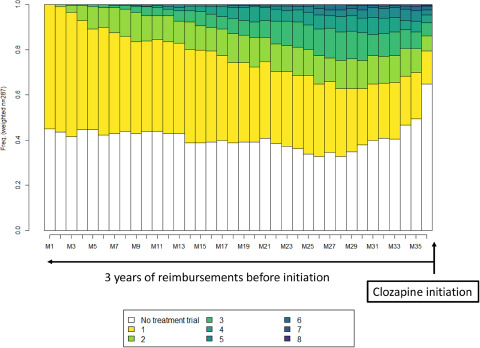
Plots of transverse distributions of different successive TTs during the 36 months before clozapine initiation for the whole population (*n* = 287).

Legend: Each month is represented on the *x*-axis from the first (M1) to the last before clozapine initiation (M36). The *y*-axis represents the percentage of individuals under successive TTs, from yellow (first TT) to dark blue (eighth TT). The white represents the absence of reimbursement for an effective treatment. The main TT represented was the first TT during the whole period.

The proportions of individuals with different antipsychotics prescribed in successive TTs during the follow-up period are shown in Fig. 2. Almost 65% of the individuals had not received any TT just before the initiation of clozapine. The addition of data on hospitalizations and the delivery of other psychotropic drugs (including noneffective antipsychotic treatments) did not cover the full proportion of individuals without TT delivery (Supplementary Material: Figure S1).

Concerning different successive TTs, the percentage of individuals in their first TT decreased from 41% (117/287) at month 12 to 15% (42/287) at month 36; the percentage of individuals who were in their second TT slightly decreased from 12% (33/287) at month 12 to 7% (19/287) at month 36; and the maximum percentage of individuals with more than two TTs was nearly 25% of the population.

### Secondary outcomes

Using TTs trajectory sequence analysis, a three-cluster typology was found with moderate quality (*ASWw* = 0.37 and *HC* = 0.08), including 133 (46.3%), 51 (17.8%) and 103 (35.9%) individuals in Clusters 1 (C1), 2 (C2) and 3 (C3), respectively. Socio-demographic characteristics, TTs and delivery data per cluster are presented in Table 2.

**Table 2.** Socio-demographic, treatment trial and delivery data of the three identified clusters

	Cluster 1 (*n* = 133)	Cluster 2 (*n* = 51)	Cluster 3 (*n* = 103)	*P* value
*Socio-demographic data*				
Sex (male), % (*n*)	56.4 (75)	70.6 (36)	61.2 (63)	0.21
Age at clozapine initiation (years), mean (sd)	40.1 (18.6)	43.0 (14.7)	46.7 (13.1)	0.008
Time since SSAD diagnosis CDS registration (years), median [IQR]	6.0 [1.0−16.0]	11.0 [4.0−20.0]	17.0 [9.5−24.0]	<0.001*
Presence of CMU-c (yes), % (*n*)	41.4 (55)	56.9 (29)	40.8 (42)	0.12
Hospitalization in psychiatric ward (yes), % (*n*)	53.4 (71)	54.9 (28)	42.7 (44)	0.19
Duration of hospitalization (days), median [IQR]	35.0 [12.5–114.0]	12.0 [4.0–44.0]	22.0 [3.0–66.0]	<0.001*
*TT data*				
Number of TTs per individual, median [IQR]	2 [1−3]	4 [3−5]	2 [1−3]	<0.001*
Duration of TT (days), median [IQR]	57.0 [29.0−105.8]	83.5 [34.0−187.3]	124.5 [57.0−321.5]	<0.001*
Individuals with >2 TTs (yes), % (*n*)	33.1% (44)	82.4% (42)	41.7% (43)	<0.0001
Individuals with at least one TT with APP (yes), % (*n*)	51.1 (68)	90.2 (46)	68.9 (71)	<0.0001
*Prescribers’ data*				
Number of prescribers, mean (sd)	3.1 (1.9)	4.0 (2.4)	3.9 (2.2)	0.003
Number of private practitioner prescriber, mean (sd)	1.2 (1.4)	1.8 (1.8)	1.6 (1.5)	0.02
Number of public health system prescribers, mean (sd)	1.9 (1.1)	2.2 (1.1)	2.3 (1.4)	0.04

Legend:

* = non-parametrical test (Kruskal–Wallis); APP = antipsychotic polypharmacy, CDS = Chronic Disease Scheme, CMU-c = Couverture maladie universelle complementaire, IQR = interquartile range, min = minimum, max = maximum, SSAD = schizophrenia or schizoaffective disorder, TT = treatment trial. Missing values: time since SSAD CDS registration = 15.7%.

**Figure 3.**
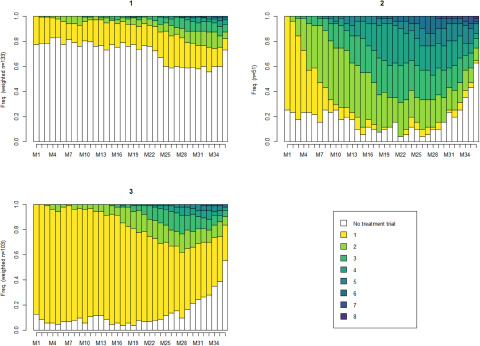
Plots of transverse distributions of different successive TTs during the 36 months before clozapine initiation by three clusters.

Legend: Each month is represented on the *x*-axis from the first (M1) to the last before clozapine initiation (M36). The *y*-axis represents the percentage of individuals under successive TTs, from yellow (first TT) to dark blue (eighth TT). The white represents the absence of reimbursement for an effective treatment. C1: *n* = 133; C2: *n* = 51, C3: *n* = 103.

The proportions of different successive TTs per cluster are shown in Fig. 3. Regarding trajectories of TTs, C1 had a lower percentage of individuals with TTs delivery, and C3 included the highest percentage. Individuals in C3 were more frequently in their first TT throughout the follow-up period. C2 contained individuals who received more than two TTs early in the follow-up period. In this cluster, the percentage of patients with more than two TTs (maximum: 8 successive TTs) delivered increased to approximately 60%. Hospitalizations and deliveries of other psychotropics in the absence of TTs were more frequent in C1; nevertheless, these factors poorly explained the absence of TTs in each cluster (Supplementary Material: Figure S2).

The sensitivity analysis on the time since CDS registration (3–10 or >10 years) did not alter the typology of the identified clusters and led to only slight variations in individual proportions (Supplementary Material: Figures S3–S6).

## Discussion

### Main results

In this study, a new method was used to evaluate the antipsychotic utilization and treatment trajectories of 287 individuals with SSAD before clozapine initiation using data from a French national database. Our results revealed (i) a median number of three TTs during the 36 months before clozapine initiation and (ii) three clusters of previous antipsychotic trajectories associated with specific individual and TT characteristics. As the average duration of illness since registration exceeded the availability of SNDS data, the number of TTs per individual was likely higher than the 2 recommended monotherapy trials. The initiation of clozapine after treatment discontinuation or hospitalization suggests either a lack of efficacy or low tolerability of the previous TT. The most frequently dispensed antipsychotics in our cohort were second-generation antipsychotics, notably risperidone. With the exception of haloperidol, first-generation antipsychotics were rarely prescribed as monotherapy. Indeed, cyamemazine and loxapine were almost exclusively dispensed as part of APP.

The same median number of antipsychotics before clozapine prescription (*n* = 3) was reported in the Wheeler study in patients with a mean duration of illness of 9.7 years (Wheeler, 2008) before clozapine initiation compared to 13 years in our study. Howes et al. reported a slightly higher mean number of antipsychotic trials (3.9) over a shorter duration of disease (8 years) before clozapine initiation (Howes *et al.*, 2012). The authors highlighted that individuals with two adequate antipsychotic trials were not prescribed clozapine until 4 years later. A shorter delay of clozapine initiation (2.5 years) with a mean number of 2.4 antipsychotic trials was also shown in a chart review (Üçok *et al.*, 2015). One factor that may explain the present results is the mean age of our sample, which was more than 10 years higher than that in previous studies (Howes *et al.*, 2012; Üçok *et al.*, 2015; Wheeler *et al.*, 2014). Indeed, older age is associated with delayed clozapine initiation (Howes *et al.*, 2012).

### Different clusters for different trajectories

We identified three different clusters of antipsychotic trajectories with significant differences in the age of individuals. Given these results, two hypotheses can be proposed. The first hypothesis is that clusters are composed of virtually the same population captured at three different times in their therapeutic pathways. In this case, a sensitivity analysis on the time since disease registration should reveal a different typology of the clusters. However, the sensitivity analysis identified the same three-cluster typology, supporting the hypothesis that these three clusters represent distinct populations with different therapeutic pathways. In C1, which contained ‘less treated’ and younger individuals, we hypothesize that the delay to clozapine initiation was associated with a concern regarding adherence. As seen in the Supplementary Materials, C1 had more individuals with other psychotropic drug deliveries in the absence of TT and had more frequent selective serotonin reuptake inhibitors deliveries than other clusters (Supplementary Materials: Table S7). Poor adherence of schizophrenia patients has been linked with younger age and depressive episodes (Misdrahi *et al.*, 2016). This may also correspond to patients with an uncertain diagnosis in early stages of the disease. C2 corresponded to ‘more treated’ individuals but less stable treatment, suggesting a lack of efficacy of successive TTs and possibly more severe psychotic symptoms. Regarding other psychotropic drugs, individuals in C2 received mood stabilizers more frequently, which may suggest frequent affective symptoms that can contribute to difficulty in obtaining an effective and stable treatment. C3 corresponded to the oldest individuals, with a longer duration of disease, more ‘treatment stable’ and less frequent hospitalizations; it can be hypothesized that clozapine initiation was motivated by extrapyramidal symptom concerns or persistence of negative symptoms.

### Discrepancies with recommendations

Contrary to recommendations, in more than two-thirds of individuals, APP was prescribed before clozapine. This is higher than the proportion in the Wheeler study (Wheeler, 2008) and roughly equivalent to that in the Üçok study (Üçok *et al.*, 2015). This could indicate an insufficient treatment of symptomatology or a delay of effective treatment because of justified or unjustified concerns about clozapine use. Wheeler showed that clozapine use was associated with a lower likelihood of APP (Wheeler, 2008). Inconsistent and debatable results on the efficacy of APP compared to monotherapy have been reported (Correll *et al.*, 2009; Galling *et al.*, 2017); in addition, in a large cohort in a study by Tiihonen et al., clozapine monotherapy showed a remarkable efficacy in reducing rehospitalizations, only three APP combinations (clozapine and aripiprazole, any LAI and olanzapine, clozapine and olanzapine) were slightly superior to it (Tiihonen *et al.*, 2019).

In a systematic review, Verdoux et al. highlighted that the main prescriber-related barriers to clozapine prescription were not doubts about its efficacy, but a lack of personal prescription experience and concern about the implementation of haematological monitoring or side effects (Verdoux *et al.*, 2018). The adherence to guidelines and polypharmacy habits of French psychiatrists also seems key, as has already been demonstrated in bipolar disorder (Bellivier *et al.*, 2014; Samalin *et al.*, 2011).

### Strengths and weaknesses

The main strength of our study is the exhaustiveness of data on antipsychotics reimbursement and TTs in a real-world setting. Our cohort is also sizeable, containing 287 individuals, which is more than previous cohorts (Brodeur *et al.*, 2022; Howes *et al.*, 2012; Sharma *et al.*, 2021; Taylor *et al.*, 2003; Üçok *et al.*, 2015; Wheeler *et al.*, 2014). While chart reviews or retrospective records can be subject to missing data or memory bias, the SNDS is a validated tool to overcome these issues (Bezin *et al.*, 2017). Although the temporal depth of SNDS data may seem limited, the follow-up period of 36 months is likely to cover two TTs of adequate duration. The methodological choice to consider TE only for antipsychotics indicated for schizophrenia with at least two successive deliveries and an average daily dose >80% of the DDD allowed us to best approach the TRRIP group’s definition of adequate treatment as ≥6 weeks at a daily chlorpromazine dose equivalent ≥600 mg (Howes *et al.*, 2017). The consideration of cyamemazine and loxapine, which are generally used for the management of acute symptoms in our methodology, is justified by the fact that two deliveries of sufficient doses indicate the need to revise antipsychotic treatment. Additionally, the use of the state sequence analysis provided a graphical representation that offered a complete and intuitive visual overview of these cross-sectional data. Brodeur *et al.* (2022) used the same methodology to study patterns of the continuation and discontinuation of antipsychotics. They also demonstrated sequence analysis is valid as an adjunct to more traditional statistical methods and complementary to the latter (as in this study).

Several methodological limitations of our study are present. First, we had no data on prescriptions during hospitalizations. This limitation is well known from observational records, as found in studies by Tiihonen (Scandinavia), Weiser (USA) and Brodeur (Canada) (Brodeur *et al.*, 2022; Tiihonen *et al.*, 2019; Weiser *et al.*, 2021). However, hospitalizations did not fully explain the absence of antipsychotic delivery. Additionally, long-acting olanzapine, which is only dispensed in facilities with medical surveillance, and long-acting antipsychotics dispensed in daytime hospitals do not appear in outpatient data (as this is not associated with an individual claim of reimbursement). Such missing data can lead to an underestimated number of TTs. Also, certain prescription strategies (such as the prescription of cyamemazine, not widely available outside France) may reflect national practices and limit the extrapolation of our data. Finally, the purpose of the study was not to see whether patients met the criteria for TRS, but it is well known that even individuals with a confirmed diagnosis of TRS may experience excessive delay before starting the most effective treatment for their condition (Howes *et al.*, 2012; Nielsen *et al.*, 2012; Wheeler, 2008). Moreover, it has been documented that some psychiatrists are reluctant to and never (or rarely) prescribe clozapine (Verdoux *et al.*, 2018).

## Conclusions

Our data revealed that patients with SSAD had a median number of antipsychotic trials higher than the recommended amount (two) during the 36 months before clozapine initiation. The use of the innovative method of state sequence analysis revealed three distinct clusters of therapeutic pathways associated with different individual characteristics. These differences allow further understanding of pre-clozapine initiation patterns. To understand the barriers to clozapine initiation and better promote its use, more user cohorts are urgently needed.

## Supporting information

Laforgue et al. supplementary material Laforgue et al. supplementary material

## Data Availability

Given the risk to the privacy of individuals, the data holder (French Health Care Insurance Institution, CNAM) has restricted the dataset from becoming publicly available. The French Institute of Health Care Data (INDS, www.indsante.fr) may be contacted with requests for data.

## Supplementary material

The supplementary material for this article can be found at https://doi.org/10.1017/S2045796023000732.
