# Supplementary material for: Characterization of antipsychotic utilization before clozapine initiation for individuals with schizophrenia: an innovative visualization of trajectories using French National Health Insurance data
Source: Epidemiol Psychiatr Sci. 2023 Sep 19;32:e59. doi: 10.1017/S2045796023000732 (PMC10539739; doi:10.1017/S2045796023000732)
Supplement: Laforgue et al. supplementary material [file S2045796023000732sup001.docx]

**Title:** Characterization of antipsychotic utilization before clozapine initiation for individuals with schizophrenia: an innovative visualization of trajectories using French National Health Insurance data

**Journal name**: Epidemiology and Psychiatric Sciences

**Running title:** Antipsychotic trajectories before clozapine

**AUTHORS INFORMATION**:

Edouard-Jules LAFORGUE, MD^(a,b)§^^[[1]](#footnote-1)^* [edouard.laforgue@chu-nantes.fr](mailto:edouard.laforgue@chu-nantes.fr) ; Marion ISTVAN, MD^(a,b)§^ [marion.istvan@chu-nantes.fr](mailto:marion.istvan@chu-nantes.fr) ; Anicet CHASLERIE, MD^(c)^ [anicet.chaslerie@assurance-maladie.fr](mailto:anicet.chaslerie@assurance-maladie.fr) ; Pascal ARTARIT, MD^(c)^ [pascal.artarit@assurance-maladie.fr](mailto:pascal.artarit@assurance-maladie.fr) ; Genevieve VALLOT, DA^(c)^ [genevieve.vallot@assurance-maladie.fr](mailto:genevieve.vallot@assurance-maladie.fr) ; Pascale JOLLIET, MD-PhD^(a,b)^ [pascale.jolliet@univ-nantes.fr](mailto:pascale.jolliet@univ-nantes.fr) ; Marie GRALL-BRONNEC, MD-PhD^(b,d)^ [marie.bronnec@chu-nantes.fr](mailto:marie.bronnec@chu-nantes.fr) ; Caroline VICTORRI-VIGNEAU, PharmD-PhD^(a,b)^ [caroline.vigneau@chu-nantes.fr](mailto:caroline.vigneau@chu-nantes.fr)

^§^Edouard-Jules Laforgue and Marion Istvan are co-first authors

^(a)^ Nantes Université, CHU Nantes, Service de Pharmacologie Clinique – Centre d’Évaluation et d’Information sur la Pharmacovigilance Addictovigilance, F-44000 Nantes, France

^(b)^ Nantes Université, CHU Nantes, INSERM, Methods in Patient-Centred Outcomes & Health Research, F-44000 Nantes, France

^(c)^ Medical Department, French Health Insurance System, 9 rue de Président Edouard Herriot, F-44000 Nantes, France

^(d)^ Nantes Université, CHU Nantes, Service d’Addictologie et de Psychiatrie de Liaison, F- 44000 Nantes, France

**Corresponding author:**

Edouard-Jules LAFORGUE

Nantes Université, CHU Nantes, Service de Pharmacologie Clinique – Centre d’Évaluation et d’Information sur la Pharmacovigilance Addictovigilance, F-44000 Nantes, France

Adress: CHU Nantes, 8 quai Moncousu, 44093 Nantes, France

[edouard.laforgue@chu-nantes.fr](mailto:edouard.laforgue@chu-nantes.fr) +33 (0)2 40 08 40 73

**Supplementary materials**

**Fig. S1** Plots of transverse TT distributions during the 36 months before clozapine initiation for the whole population with hospitalizations and other psychotropic drugs deliveries (*n* = 287)

**
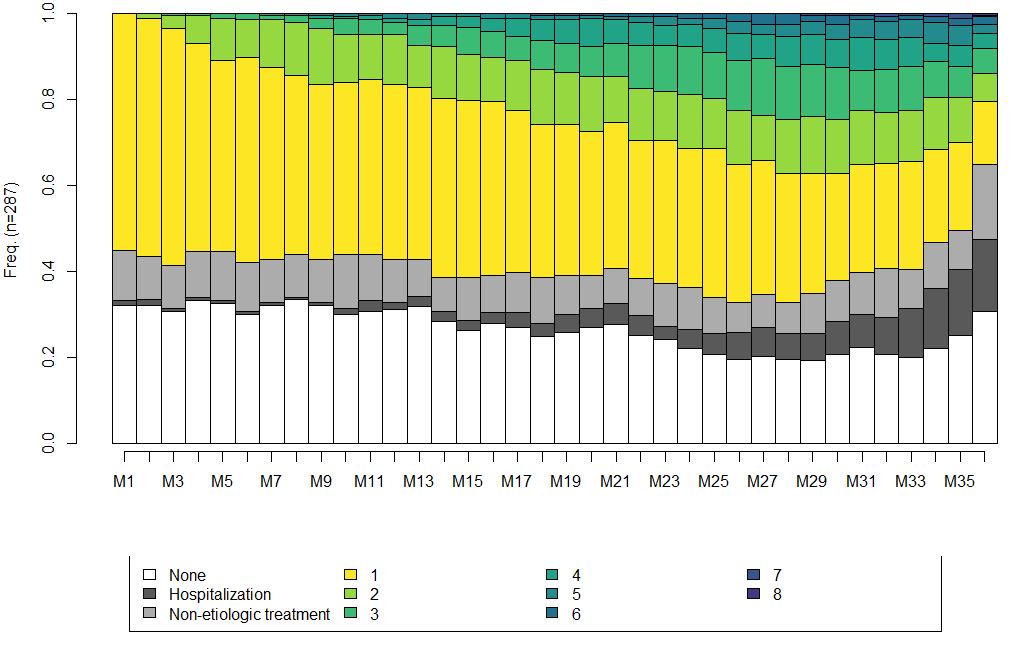
**


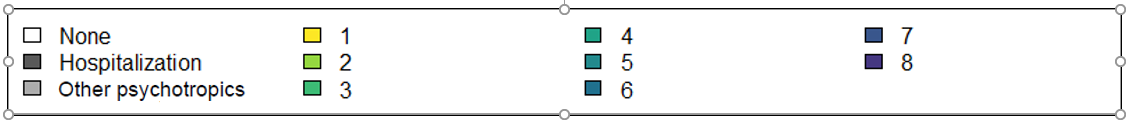


*Legend: Each month prior to clozapine initiation is represented on the x-axis from the first month of the follow-up period (M1 - left) to the last month before clozapine initiation (M36 – right). The y-axis represents the percentage of individuals under successive TTs, from yellow (1st TT) to dark blue (8th TT). Dark grey represents the percentage per month of hospitalized individuals, and clear grey represents the percentage of individuals with deliveries of* other psychotropic drugs *treatment without a TT. The white represents the absence of reimbursement for an effective treatment.*

**Fig. S2** Plots of transverse TT distributions during the 36 months before clozapine initiation with hospitalizations and other psychotropic drugsby 3 clusters


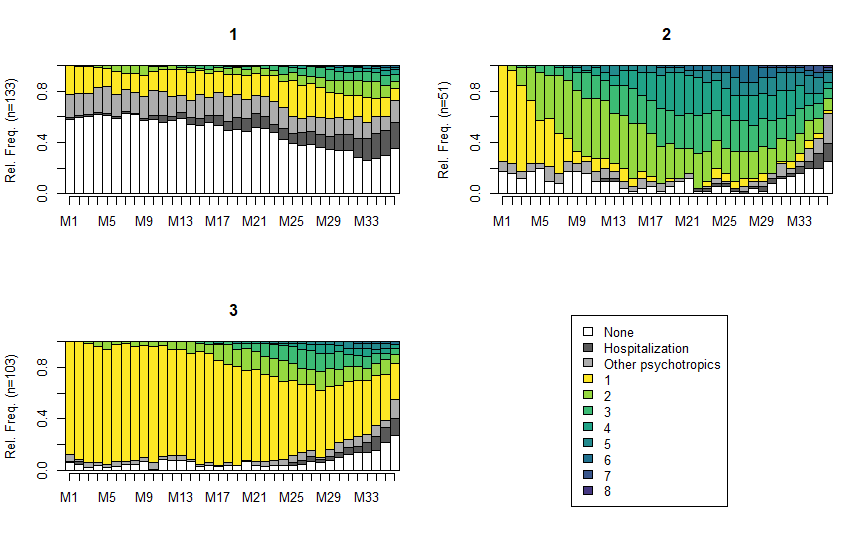


*Legend: For each cluster, each month prior to clozapine initiation is represented on the x-axis from month 1 (M1) to the last month (M36) prior to clozapine initiation. The y-axis represents the percentage of individuals under successive TTs, from yellow (1st TT) to dark blue (8th TT). The white represents the absence of reimbursement for an aetiologic treatment. For Cluster 1: n = 133; Cluster 2: n = 51, Cluster 3: n = 103. Dark grey represents the percentage per month of hospitalized individuals, and clear grey represents the percentage of individuals with deliveries of non-effective treatment without a TT. The white represents the absence of reimbursement for an effective treatment.*

**Fig. S3** Plots of transverse TT distributions during the 36 months before clozapine initiation for the population between 3 and 10 years after disease registration (*n* = 79)


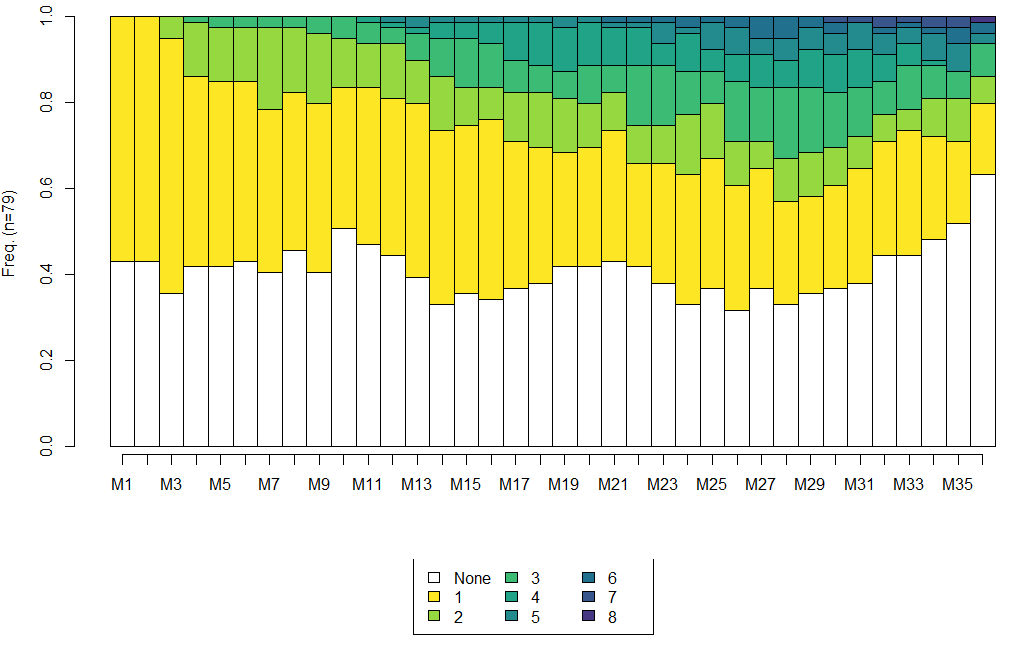


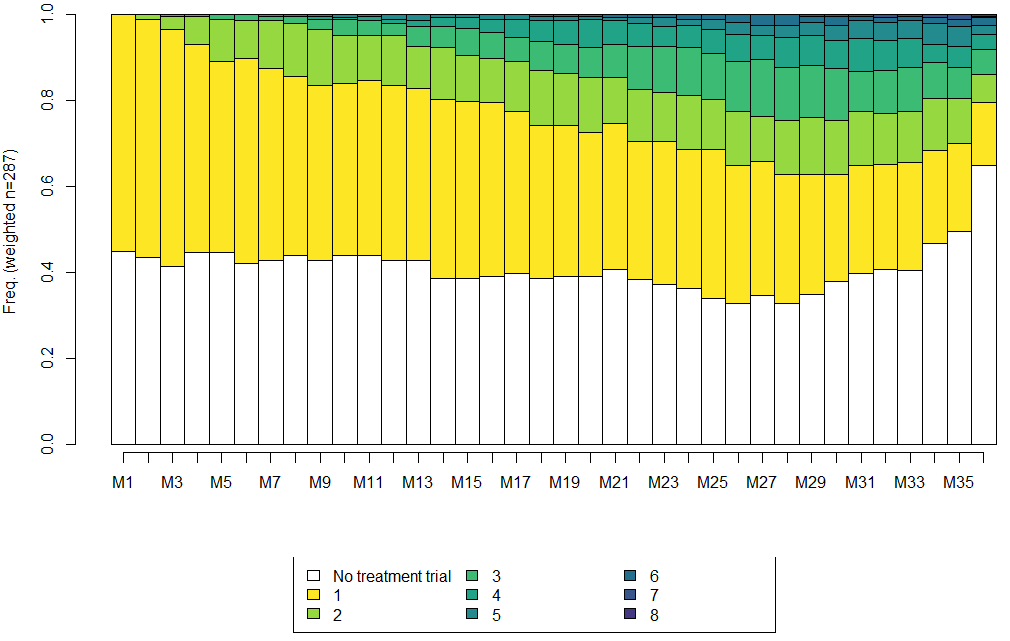


*Legend: Each month prior to clozapine initiation is represented on the x-axis from month 1 (M1) to the last month (M36) prior to clozapine initiation. The y-axis represents the percentage of individuals under successive TTs, from yellow (1st TT) to dark blue (8th TT). The white represents the absence of reimbursement for an effective treatment.*

**Fig. S4** Plots of transverse TT distributions during the 36 months before clozapine initiation for the population between 3 and 10 years since disease registration by 3 clusters


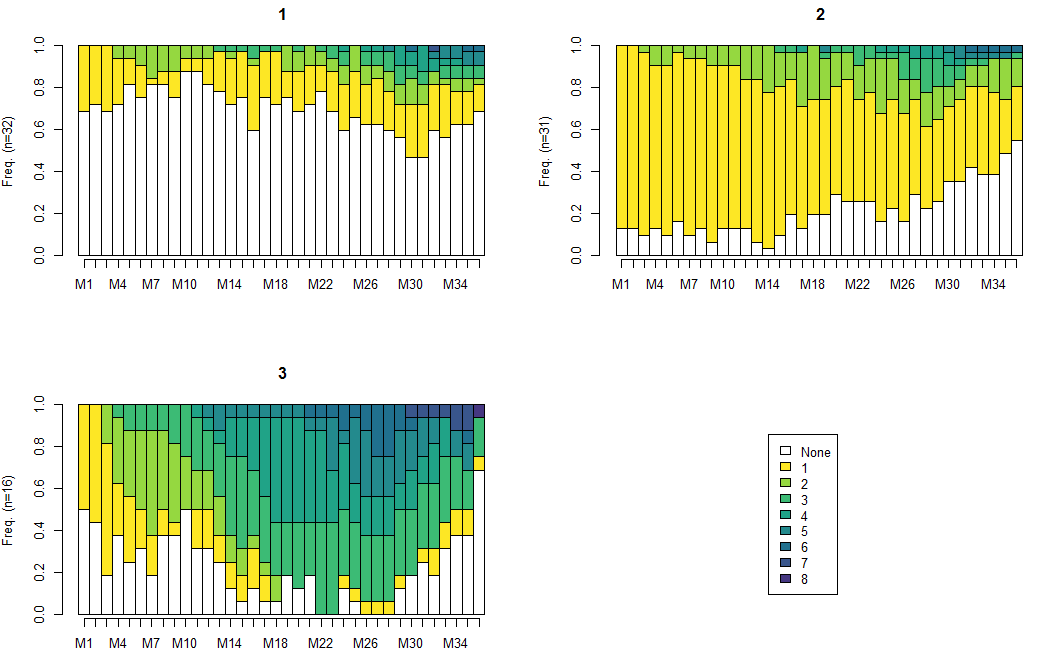


*Legend: For each cluster, each month prior to clozapine initiation is represented on the x-axis from month 1 (M1) to the last month (M36) prior to clozapine initiation. The y-axis represents the percentage of individuals under successive TTs, from yellow (1st TT) to dark blue (8th TT). The white represents the absence of reimbursement for an effective treatment. For Cluster 1: n = 32/79 (41%); Cluster 2: n = 31/79 (39%), Cluster 3: n = 16/79 (20%).*

**Fig. S5** Plots of transverse TT distributions during the 36 months before clozapine initiation for the population over 10 years after disease registration (*n* = 123)


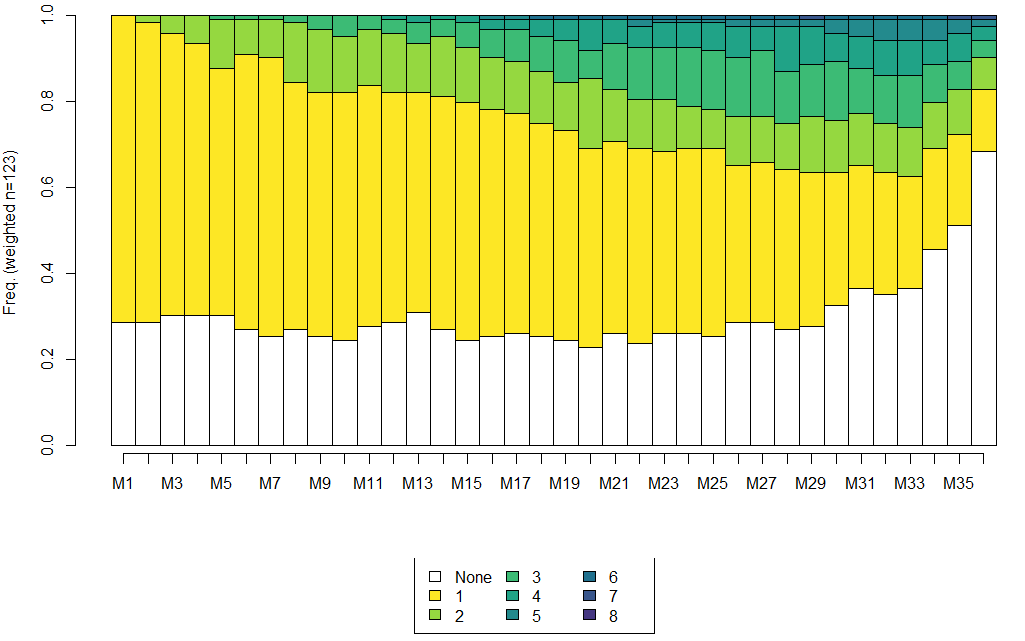

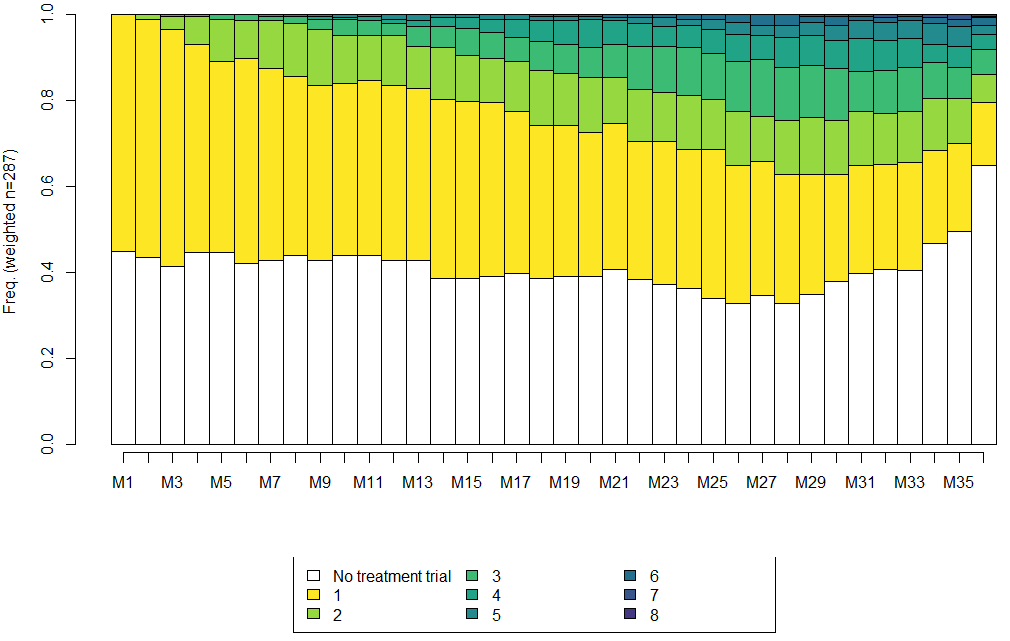


*Legend: Each month prior to clozapine initiation is represented on the x-axis from month 1 (M1) to the last month (M36) prior to clozapine initiation. The y-axis represents the percentage of individuals under successive TTs, from yellow (1st TT) to dark blue (8th TT). The white represents the absence of reimbursement for an* effective *treatment.*

**Fig. S6** Plots of transverse TT distributions during the 36 months before clozapine initiation for the population over 10 years since disease registration by 3 clusters


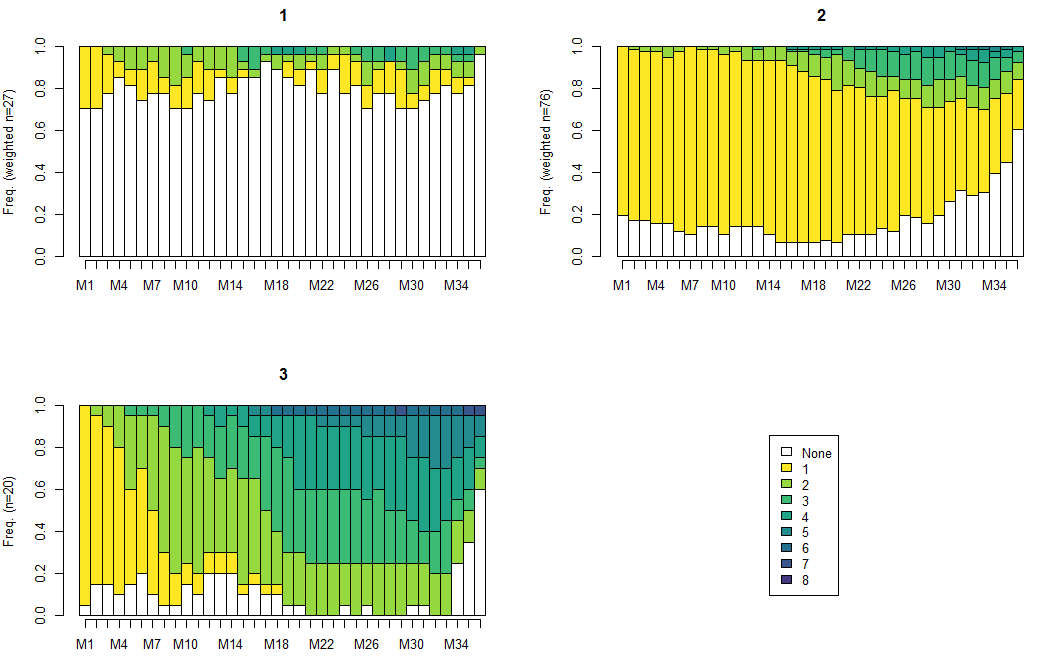


*Legend: For each cluster, each month prior to clozapine initiation is represented on the x-axis from month 1 (M1) to the last month (M36) prior to clozapine initiation. The y-axis represents the percentage of individuals under successive TTs, from yellow (1st TT) to dark blue (8th TT). The white represents the absence of reimbursement for an effective treatment. For Cluster 1: n = 27/123 (22%); Cluster 2: n = 76/123 (62%), Cluster 3: n = 20/123 (16%).*

**Table S7**. Details of other psychotropic drugs dispensed (at least one dispensation) during the 36 months before clozapine initiation for the three clusters.

Cluster 1 Cluster 2 Cluster 3

(n = 129) (n = 51) (n = 101)

ACAMPROSATE = yes (%) 1 ( 0.8) 0 ( 0.0) 0 ( 0.0)

AGOMELATINE = yes (%) 0 ( 0.0) 1 ( 2.0) 1 ( 1.0)

ALPRAZOLAM = yes (%) 38 (29.5) 18 (35.3) 30 (29.7)

AMITRIPTYLINE = yes (%) 5 ( 3.9) 1 ( 2.0) 10 ( 9.9)

BIPERIDENE = yes (%) 6 ( 4.7) 2 ( 3.9) 5 ( 5.0)

BROMAZEPAM = yes (%) 11 ( 8.5) 2 ( 3.9) 14 (13.9)

BUPRENORPHINE = yes (%) 0 ( 0.0) 3 ( 5.9) 2 ( 2.0)

BUSPIRONE = yes (%) 1 ( 0.8) 0 ( 0.0) 1 ( 1.0)

CARBAMAZEPINE = yes (%) 5 ( 3.9) 2 ( 3.9) 1 ( 1.0)

CHLORPROMAZINE = yes (%) 2 ( 1.6) 1 ( 2.0) 2 ( 2.0)

CITALOPRAM = yes (%) 2 ( 1.6) 1 ( 2.0) 3 ( 3.0)

CLOBAZAM = yes (%) 3 ( 2.3) 2 ( 3.9) 3 ( 3.0)

CLOMIPRAMINE = yes (%) 4 ( 3.1) 4 ( 7.8) 4 ( 4.0)

CLONAZEPAM = yes (%) 3 ( 2.3) 1 ( 2.0) 0 ( 0.0)

CLORAZEPATE POTASSIQUE = yes (%) 19 (14.7) 8 (15.7) 9 ( 8.9)

CYAMEMAZINE = yes (%) 31 (24.0) 19 (37.3) 40 (39.6)

DIAZEPAM = yes (%) 35 (27.1) 13 (25.5) 30 (29.7)

DISULFIRAM = yes (%) 0 ( 0.0) 1 ( 2.0) 1 ( 1.0)

DOXEPINE = yes (%) 1 ( 0.8) 0 ( 0.0) 1 ( 1.0)

DULOXETINE = yes (%) 9 ( 7.0) 4 ( 7.8) 1 ( 1.0)

ESCITALOPRAM = yes (%) 19 (14.7) 7 (13.7) 11 (10.9)

ESTAZOLAM = yes (%) 0 ( 0.0) 0 ( 0.0) 2 ( 2.0)

ETHYLE LOFLAZEPATE = yes (%) 0 ( 0.0) 0 ( 0.0) 1 ( 1.0)

ETIFOXINE = yes (%) 1 ( 0.8) 0 ( 0.0) 1 ( 1.0)

FLUOXETINE = yes (%) 6 ( 4.7) 2 ( 3.9) 2 ( 2.0)

GABAPENTINE = yes (%) 1 ( 0.8) 0 ( 0.0) 2 ( 2.0)

HYDROXYZINE = yes (%) 16 (12.4) 5 ( 9.8) 14 (13.9)

LAMOTRIGINE = yes (%) 6 ( 4.7) 6 (11.8) 4 ( 4.0)

LEVETIRACETAM = yes (%) 1 ( 0.8) 2 ( 3.9) 1 ( 1.0)

LEVOMEPROMAZINE = yes (%) 5 ( 3.9) 4 ( 7.8) 10 ( 9.9)

LITHIUM = yes (%) 12 ( 9.3) 9 (17.6) 7 ( 6.9)

LOPRAZOLAM = yes (%) 2 ( 1.6) 1 ( 2.0) 2 ( 2.0)

LORAZEPAM = yes (%) 15 (11.6) 6 (11.8) 7 ( 6.9)

LORMETAZEPAM = yes (%) 14 (10.9) 8 (15.7) 13 (12.9)

LOXAPINE = yes (%) 55 (42.6) 27 (52.9) 40 (39.6)

METHADONE = yes (%) 0 ( 0.0) 0 ( 0.0) 1 ( 1.0)

MIANSERINE = yes (%) 11 ( 8.5) 4 ( 7.8) 9 ( 8.9)

MILNACIPRAN = yes (%) 0 ( 0.0) 0 ( 0.0) 1 ( 1.0)

MIRTAZAPINE = yes (%) 17 (13.2) 5 ( 9.8) 8 ( 7.9)

NALMEFENE = yes (%) 2 ( 1.6) 1 ( 2.0) 1 ( 1.0)

NALTREXONE = yes (%) 1 ( 0.8) 1 ( 2.0) 1 ( 1.0)

NICOTINE = yes (%) 5 ( 3.9) 6 (11.8) 11 (10.9)

NORDAZEPAM = yes (%) 1 ( 0.8) 0 ( 0.0) 0 ( 0.0)

OXAZEPAM = yes (%) 24 (18.6) 16 (31.4) 18 (17.8)

OXCARBAZEPINE = yes (%) 0 ( 0.0) 1 ( 2.0) 0 ( 0.0)

PAROXETINE = yes (%) 16 (12.4) 6 (11.8) 11 (10.9)

PIPAMPERONE = yes (%) 2 ( 1.6) 0 ( 0.0) 0 ( 0.0)

PRAZEPAM = yes (%) 10 ( 7.8) 3 ( 5.9) 9 ( 8.9)

PREGABALINE = yes (%) 2 ( 1.6) 1 ( 2.0) 2 ( 2.0)

PRIMIDONE = yes (%) 1 ( 0.8) 0 ( 0.0) 0 ( 0.0)

PYRIDOSTIGMINE = yes (%) 0 ( 0.0) 0 ( 0.0) 2 ( 2.0)

RIVASTIGMINE = yes (%) 1 ( 0.8) 0 ( 0.0) 0 ( 0.0)

SERTRALINE = yes (%) 13 (10.1) 4 ( 7.8) 2 ( 2.0)

SULPIRIDE = yes (%) 1 ( 0.8) 0 ( 0.0) 0 ( 0.0)

TETRABENAZINE = yes (%) 0 ( 0.0) 1 ( 2.0) 0 ( 0.0)

TIANEPTINE = yes (%) 1 ( 0.8) 0 ( 0.0) 0 ( 0.0)

TIAPRIDE = yes (%) 1 ( 0.8) 2 ( 3.9) 0 ( 0.0)

TOPIRAMATE = yes (%) 0 ( 0.0) 1 ( 2.0) 0 ( 0.0)

TRIHEXYPHENIDYL = yes (%) 30 (23.3) 17 (33.3) 25 (24.8)

TROPATEPINE = yes (%) 63 (48.8) 24 (47.1) 46 (45.5)

VALPROIQUE ACIDE = yes (%) 20 (15.5) 17 (33.3) 15 (14.9)

VALPROMIDE = yes (%) 10 ( 7.8) 6 (11.8) 17 (16.8)

VENLAFAXINE = yes (%) 14 (10.9) 10 (19.6) 16 (15.8)

VORTIOXETINE = yes (%) 2 ( 1.6) 2 ( 3.9) 5 ( 5.0)

ZOLPIDEM = yes (%) 24 (18.6) 3 ( 5.9) 11 (10.9)

ZOPICLONE = yes (%) 44 (34.1) 18 (35.3) 35 (34.7)

ZUCLOPENTHIXOL = yes (%) 15 (11.6) 6 (11.8) 6 ( 5.9)

1. * Corresponding author [↑](#footnote-ref-1)
